# A Framework for Evaluating Field-Based, High-Throughput Phenotyping Systems: A Meta-Analysis

**DOI:** 10.3390/s19163582

**Published:** 2019-08-17

**Authors:** Sierra N. Young

**Affiliations:** Department of Biological and Agricultural Engineering, North Carolina State University, Raleigh, NC 27695, USA; syoung2@ncsu.edu; Tel.: +1-919-515-6740

**Keywords:** systems analysis, human-machine interaction, complexity analysis, technology adoption

## Abstract

This paper presents a framework for the evaluation of system complexity and utility and the identification of bottlenecks in the deployment of field-based, high-throughput phenotyping (FB-HTP) systems. Although the capabilities of technology used for high-throughput phenotyping has improved and costs decreased, there have been few, if any, successful attempts at developing turnkey field-based phenotyping systems. To identify areas for future improvement in developing turnkey FB-HTP solutions, a framework for evaluating their complexity and utility was developed and applied to total of 10 case studies to highlight potential barriers in their development and adoption. The framework performs system factorization and rates the complexity and utility of subsystem factors, as well as each FB-HTP system as a whole, and provides data related to the trends and relationships within the complexity and utility factors. This work suggests that additional research and development are needed focused around the following areas: (i) data handling and management, specifically data transfer from the field to the data processing pipeline, (ii) improved human-machine interaction to facilitate usability across multiple users, and (iii) design standardization of the factors common across all FB-HTP systems to limit the competing drivers of system complexity and utility. This framework can be used to evaluate both previously developed and future proposed systems to approximate the overall system complexity and identify areas for improvement prior to implementation.

## 1. Introduction

While there has been much work and progress in developing automated, commercial phenotyping systems in controlled environments (e.g., LemnaTech Conveyor Scanalyzer, Phenospex TraitFinder), there remains a need to continue developing systems for phenotyping at larger field scales. Field-based systems that employ proximal sensing approaches enable data collection at high spatial resolutions necessary for measuring a variety of morphological and physiological traits in realistic growing conditions over an entire growing season. These FB-HTP systems commonly utilize image, spectral, and climate sensors to collect data at the plant, row, or plot level in crop systems, operating with varying levels of autonomy. These field systems are typically built by interdisciplinary academic research teams for highly specialized phenotyping needs. Although the capabilities of technology used for high-throughput phenotyping have improved and costs decreased, there have been few, if any, successful attempts at developing turnkey field-based phenotyping systems. To address this issue, this work presents and implements a framework for characterizing system utility and complexity and evaluates FB-HTP systems through a meta-analysis to identify bottlenecks in adoption, and identifying areas for future work in system improvement.

It has been suggested that resources for developing these systems, maintaining standards for data collection and management, integrating data from multiple sources, environmental challenges, and the need for specialized solutions may all be limiting factors of FB-HTP development. The phenomics community has acknowledged difficulties due the complexity and scale of data needed by the academic sector and breeding programs [1,2]. Additionally, it has been observed that FB-HTP platforms are built by large, interdisciplinary teams for specialized needs and require significant resources [3], prompting recommendations for phenotyping systems to be low cost [3,4], usable [2,3], and open source [1,2,3], but unfortunately these recommendations have yet to be adopted across a wide range of field-based phenotyping applications. There remains a lack of development and adoption of turnkey FB-HTP systems, particularly ground-based systems, suggesting that a systematic investigation into development challenges is warranted.

**Figure 1:**
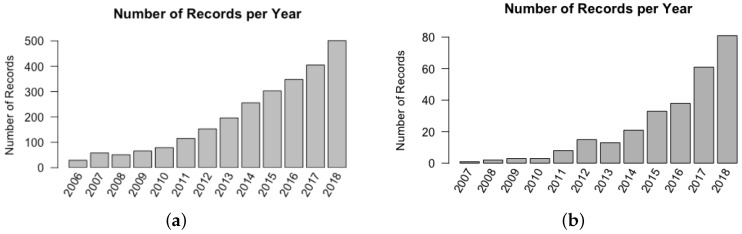
(**a**) The total number of records matching search criteria for “field AND phenotyping”, and (**b**) total number of records matching search criteria for “field AND phenotyping AND high throughput”. To eliminate irrelevant topics, search results were filtered to include the following fields: agriculture, plant sciences, science technology other topics, imaging science photographic technology, computer science, engineering, instrumentation, remote sensing, automation control systems, and robotics.

To motivate the need of understanding bottlenecks in developing FB-HTP systems, a citation search using the Web of Science database was conducted to understand recent trends of field-based phenotyping systems in the literature. The search queried all records with the words “field AND phenotyping” present in the title, abstract, or keywords which resulted in 2823 records. A second search was similarly conducted and filtered using the terms “field AND phenotyping AND high throughput” to focus on the development of HTP systems, which resulted in 299 records. It can be easily seen from Figure 1 that the number of publication records focusing on field-based phenotyping systems has steadily increased over the past decade, indicating a trend of increased efforts in development, evaluation, and deployment of this technology. Although work in FB-HTP development is temporally increasing, this field is likely still in its infancy, and a framework for conducting systems-level analysis is merited to identify potential areas of technology advancement and adoption as this trend continues.

The purpose of developing an evaluation framework is to assess the quality, performance, and sophistication of the system [5]. The development of a framework also enables the evaluation of the intrinsic properties, usability, and utility of FB-HTP systems in their environment. Related work has been done to develop methods of evaluating usability of agent-based information systems [6], including methods which decompose a central domain into smaller dimensions and use qualitative scales to determine ratings based on a set of predetermined criteria [7,8]. Comparatively, a systems approach can also assess subsystem properties to evaluate emergent properties of the system in its entirety [9] and evaluate interactions between subsystems [10]. These types of analysis tools are useful for FB-HTP systems due to their system complexity, multiple and diverse users, and the necessity to perform system decomposition for cross-platform analysis and comparison.

One particularly useful framework for software evaluation developed by Boloix and Pierre [5] enabled assessment of the system’s quality and sophistication by investigating and consolidating multiple points of view. Additionally, their framework simplified complex mechanisms to make large amounts of available information useful and provide a basis for comparison. Their framework [5] was adapted for FB-HTP platforms by taking a systems approach and integrating it with an approach for project complexity evaluation [11,12]. This newly developed framework provides a tool for evaluating FB-HTP systems that considers the following points of view: the research project goals, the phenotyping platform requirements, the crop system requirements, and the data analysis pipeline. The evaluation outcome is valuable because it identifies gaps in current research efforts and points to future research directions that are necessary to overcome the current challenges related to developing turnkey hardware and software for FB-HTP platforms.

This paper is organized as follows: Drawing on insights from the literature and a qualitative study, Section 2 presents the systems approach and framework development for evaluating FB-HTP systems. Section 3 describes the implementation of the framework on a set of 10 field-based phenotyping systems and includes results from the framework evaluation. Finally, Section 4 presents a discussion of the results, and Section 5 presents the conclusions and future directions of this work.

## 2. The Framework and Evaluation Approach

The original Boloix and Pierre framework was built on the Goal Question Metric (GQM) approach, which assumes that goals are specified first, then the data required to complete those goals are identified, followed by providing a framework for handling and interpreting the data in order for a system to operate in a purposeful way [13]. The adaptation of their framework to FB-HTP systems required taking a systems approach, which considers the individual attributes of an entire system to achieve the overall objective of a system. For this study, the overall system objective is to perform high-throughput phenotyping in a field environment with a system that, from the perspective of a user, is easy to use (less complex) and has the most added value (highest utility). This approach evaluates the phenotyping system as a whole by decomposing it into appropriate subsystem dimensions and performing factorization to assess complexity and utility at the smallest, common level. By conducting an assessment at the subsystem and factor levels, complex behaviors can be managed and captured, and relationships between subsystems and emergent behaviors of the whole system become apparent.

### 2.1. Defining the System Boundary

**Figure 2:**
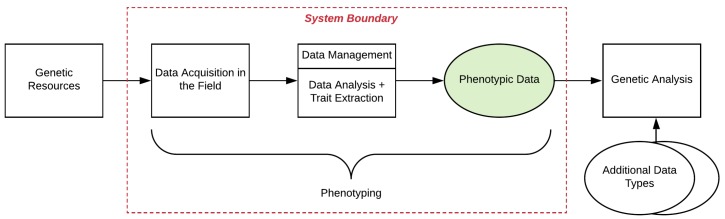
The red dotted line highlights the system boundary of the field-based, high-throughput phenotyping (FB-HTP) systems included in this analysis.

There are many steps in the overall breeding process, where FB-HTP phenotyping plays only a sub-role, albeit a critical one. The system boundary considered in this framework is shown in Figure 2 below. The FB-HTP process takes place after the genetic resources and plant material are in place in a field environment, and is considered terminated when the phenotypic traits have been extracted and are ready for integration with other data to perform genetic analysis. These components, and their corresponding subsystems and factors as described in the subsequent sections, are considered for analysis within this framework.

### 2.2. Defining the Elements

**Figure 3:**
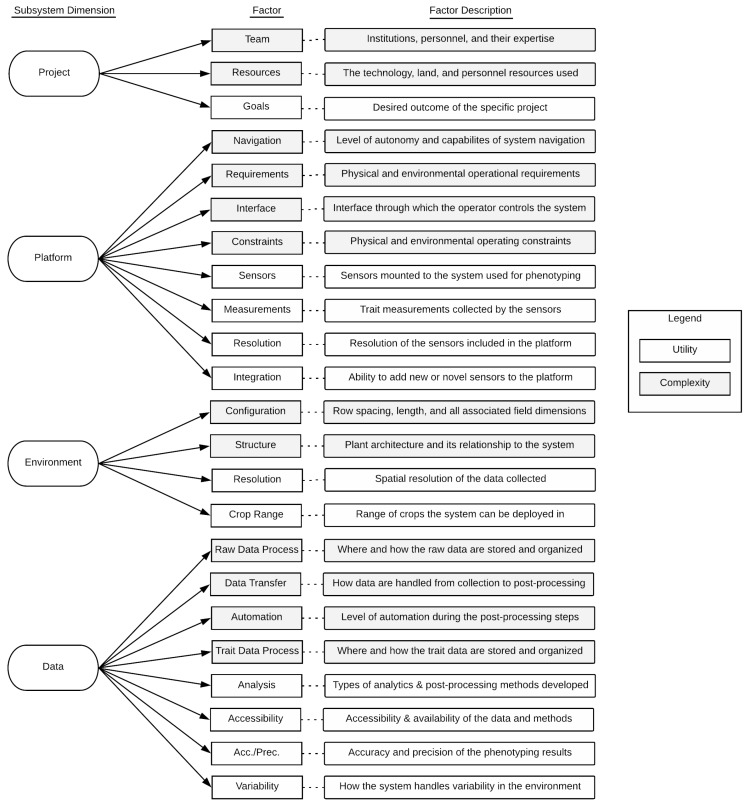
Illustration of the subsystem dimensions, factors, and their relationships included in this framework. A brief description of each factor is included in the panels on the right. Definitions for each subsystem and factor are included in Section 2.2. Factors in the gray shaded boxes are associated with system utility, and factors in white boxes are associated with system complexity.

A complete phenotyping system is made up of subsystems, or smaller units, that are interconnected and have inputs and outputs. The subsystem dimensions in this framework are integral for considering the complete phenotyping system and include the following: the project components, the physical system, the operating environment, and the resulting data and processing pipeline. These subsystems provide different opportunities for evaluation, and depending on the project team, roles may be allocated according to the subsystems. For each given subsystem there are a set of factors determined by further decomposition which represent the smallest unit that can effectively be analyzed and compared across different phenotyping systems. These factors, depending on their function, contribute differently to the overall operation of the system; some factors affect the usability of the system, or its *complexity* (which should be minimized), while some factors affect the overall value obtained from using the system, or the *utility* (which should be maximized). The factors can be categorized according to their complexity and utility contributions, which are useful attributes for classification from maturity and usability perspectives [5]. The major subsystems and their associated factors that are common to all FB-HTP platforms are illustrated in Figure 3. It is important to note that the complexity rating is determined from the perspective of the user or operator, as opposed to the developer. For example, a fully autonomous navigation system may have the most complex source code, but would be easier for someone to use than a system that requires continuous manual input for operation. Each subsystem, the associated factors, and category ratings are defined and described in greater detail in the following sections.

#### 2.2.1. Project

The project subsystem characterizes the context of the FB-HTP project, including the team (personnel and institutions), goals, and resources available for completion. These factors guide the project and provide a top-down approach for determining the subsequent subsystems and associated factors. Table 1 describes the team and resource complexity factors, and the goal utility factor, and provides descriptions for the *Basic*, *Intermediate*, and *Advanced* categories from a user’s perspective.

#### 2.2.2. Platform

The platform subsystem characterizes the machine properties, accompanying sensors, and resulting capabilities of the FB-HTP system with a focus on the physical platform. The complexity factors associated with this subsystem include navigation, operational requirements, user interface, and operational constraints. The utility factors include the sensors, possible phenotypic measurements, sensor resolution, and ability to integrate new sensors. All factors are detailed in Table 2 below.

#### 2.2.3. Environment

The environment subsystem characterizes the operational environment (e.g., the crop system) of the phenotyping platform. Specific complexity factors associated with the environmental dimension include planting configuration and the plant structure, and utility factors include the environmental resolution of the data and the range of crops suitable for deployment, which are described in Table 3.

#### 2.2.4. Data

The data subsystem characterizes the resulting data collected from the platforms and the processing pipeline, encompassing the entire data handling process. Specific factors associated with this subsystem’s complexity include raw data management, data transfer methods, the level of post-processing automation, and trait data management. Utility factors include the types of analysis developed, the level of accessibility of the trait analysis methods, the accuracy of the methods, and how the system accounts for variability in environmental conditions in the resulting data. All factors are described in Table 4.

## 3. Applying the Framework

### 3.1. Selection of Case Studies

A total of 10 FB-HTP system case studies were selected from the literature that met the following criteria: (i) the phenotyping system was built for high-throughput field deployment; (ii) the reference(s) included information about the platform (including development, sensors, and operation), and included some examples of phenotypic data and analysis, and (iii) the deployment scale was appropriate for breeding trials (i.e., production agriculture technology was not included). Information about the case studies is included in Table 5, including the participating institutions, project goals, platform type, and crop system that the platform was deployed or tested in. Detailed technical information of each case study is not included for brevity, although this information can be found in the Supplementary Materials.

### 3.2. Applying the Framework and Results

To apply this framework, each factor within the subsystems of all 10 case studies was evaluated against the factor criteria presented in Table 1, Table 2, Table 3 and Table 4. All information considered for this analysis was included in the provided references. When not enough information was available in the reference, the designation “NA” or “not available” was given. The complexity and utility ratings were reported on the following five-point scale: 1 = *Basic*, 2 = *Basic-Intermediate*, 3 = *Intermediate*, 4 = *Intermediate-Advanced*, and 5 = *Advanced*. The ratings provided are from the perspective of the user, meaning they would value a highly advanced system for the utility ratings, but would prefer a lower rating (less advanced) for the complexity ratings. The values were recorded in a table format similar to the Crawford-Ishikura Factor Table for Evaluating Roles (CIFTER) presented in [11,12]. The factor complexity scores and utility scores were added together to determine a total complexity and utility score for each FB-HTP platform, respectively. Detailed results from applying this framework for each study are included in the Supplementary Materials, and the final scoring results are included in Section 3.3 below.

### 3.3. Results of Framework Application

#### 3.3.1. Complexity Scoring

The system complexity scoring results are shown in Table 6. As can be seen from the values in the table, there is a wide range of complexity levels across subsystems and factors, as well as within subsystems for a given phenotyping platform. The project team factor had complexity ratings of *intermediate* or higher for half of the systems (n=5), and the systems that had lower complex ratings were teams comprised primarily of personnel from the same institution or from only engineering disciplines (n=5) [19,21,23,24,25]. The resources factor ratings were highly variable, which is to be expected as each intuition and project has their own available equipment, field locations, and facilities, and there were no significant correlations between the resources factor and any other factor included in the complexity analysis.

All of the FB-HTP systems required some level of human input for navigation. A total of n=5 systems were considered highly autonomous, while n=5 of the systems required more continuous manual inputs. One trend for the interface implementation was the use of a graphical user interface, or GUI, for user input, although a few of the systems did not explicitly address the human machine interface technology [16,20,25]. The system operational requirements and constraint ratings were also variable, although complex operational requirements were normally associated with complex constraints [14,19,20,21]. In addition, complex sensor configurations enabled more complex measurements to be collected [14,16,24,25], which is as expected. A few operating environments were considered to be moderately complex [14,20,22], largely due to the height and density of the crop and the row spacing, making measurements of individual plants challenging. A majority of systems operated in intermediate or basic environments, with adequate row spacing to fit larger equipment (for example, [15]), or planting configurations that facilitate measurements at the individual plant level (for example, [24]). A total of n=3 systems implemented automated organization of raw data into databases located on board servers during the data collection processes [14,15,16]; however, this was not feasible for some systems due to their form factor, which could not house the same types of computing systems (for example, [19,20]). There were also trends in a lack of non-reported information related to trait data management and storage aspects of the system. While reporting on data handling methods may not be of scientific interest for documentation purposes in all cases, it is a critical component that ultimately affects the usability and complexity of these systems.

**Figure 4:**
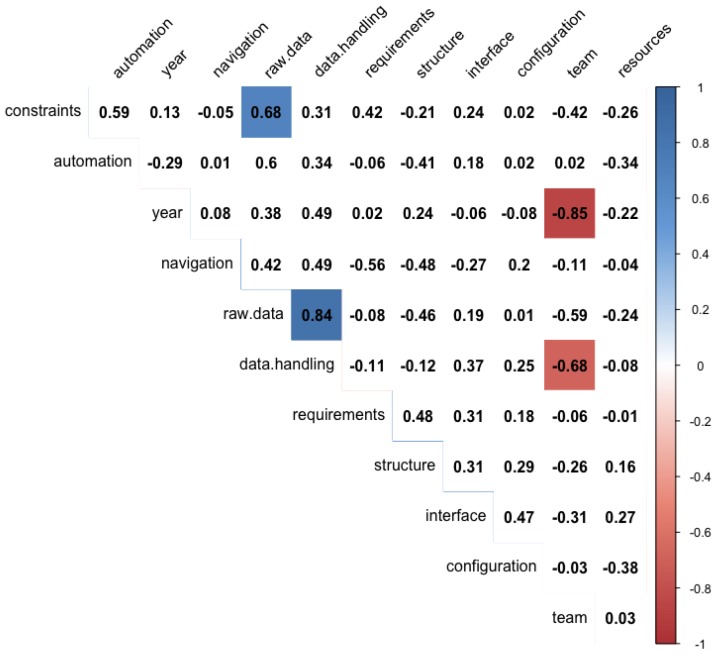
The intercorrelations between the complexity scores for the subsystem factors. Only scores that were significant at the p<0.05 level were shaded in the matrix. Note that the trait data management factor was not included in this correlation analysis due to the high number of missing data points, as can be seen from Table 6.

**Figure 5:**
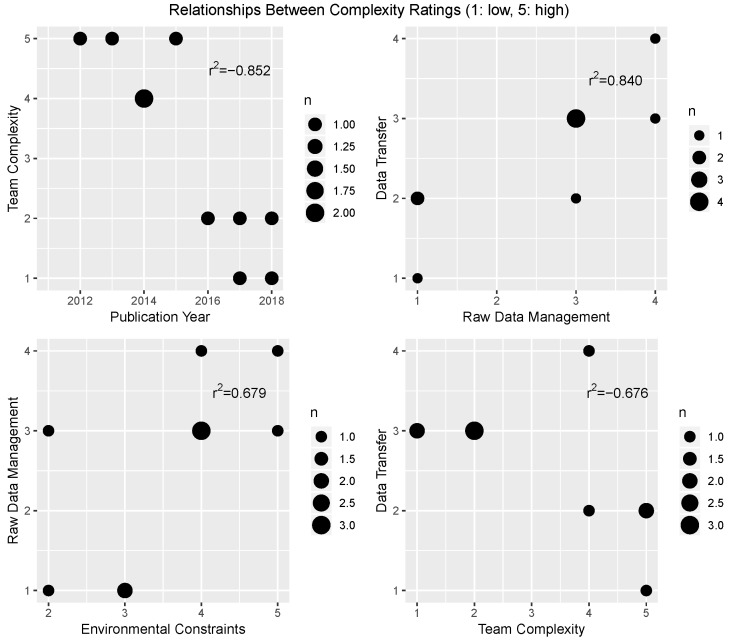
Scatter plots of the significant complexity correlations from the analysis presented in Figure 4: Team complexity and publication year ($r^{2}=-0.852$); raw data management and data transfer ($r^{2}=0.840$); raw data management and environmental constraints ($r^{2}=0.679$); and data transfer and team complexity ($r^{2}=-0.676$).

A correlation matrix for the complexity ratings is shown in Figure 4, with only the significant correlations (p<0.05) highlighted. Due to the data being ordinal in nature, the Spearman correlation coefficients were calculated and are shown in the matrix. In general, the phenotyping platform complexity is not directly dependent on any single factor, but rather is dependent on many factors; however, there were several significant correlations between individual complexity factors. For further exploration of these trends, scatter plots for these data are shown in Figure 5. The first plot indicates that more recent publications on the design and evaluation of FB-HTP systems include teams that are less complex, or teams that have fewer individuals representing fewer disciplines. This may be an indicator of increased interest and work in the phenotyping domain from individuals with an engineering background, or a shift towards a more narrow focus into the technology development of more advanced robotic systems. Additionally, it is important to note the authorship of these reference materials does not necessarily represent all personnel who may have contributed to the system development overall, and it is likely that all of these systems were the results of interdisciplinary efforts (as sometimes evidenced by the acknowledgement sections). There is a second trend that systems with less complex raw data management strategies (e.g., automatic storage and labeling processes) resulted in less complex data handling and transfer methods across the post-processing pipeline, possibly indicating that development effort up front in data management may reduce the effort in handling these data throughout the rest of the project. There is also a trend that systems with more complex environmental constraints had more complex raw data management strategies, perhaps because the platforms designed to handle more complex environments, such as small mobile robots [19,20,25], cannot carry the same industrial PCs and servers that large sensor carts of tractor systems can (however, those larger systems are typically less flexible in their deployment environments). Finally, there was a moderate negative linear relationship between the data transfer methods and the team complexity, which perhaps indicates that teams with more diverse expertise were better equipped with the knowledge and expertise to design the sub-systems and processes within a FB-HTP system, although this was not evident from the remainder of the relationships between team complexity and the other complexity factors.

#### 3.3.2. Utility Scoring

The factory utility scoring results are shown in Table 7. Similarly to the complexity rating results, there is a wide range of complexity levels across subsystems and factors, as well as within subsystems for a given phenotyping platform. The goal factor ratings were highly variable, which is to be expected as each intuition and project has their own agenda for conducting the associated research. Three total systems were rated the highest utility score for the number of sensors used [14,16,24], which also corresponded to high utility ratings for the possible phenotypes measured. There were a few systems who used fewer sensors to collect measurements with higher utility, specifically the systems developed in [21,25] which were able to create 3D reconstructions of the plants, which are highly valuable for obtaining plant architecture traits, using concepts of stereovision and relatively simple sensors (e.g., RGB cameras). Several systems that scored high for sensor integration utility were designed to be flexible and modular from the start [16,24,25]. For the environment factors, systems that enabled data collection at the individual plant level scored highest for environmental resolution. Platforms that had more flexibility in deployment (with the UAV ranking highest, and systems with flexible wheelbases or between-row systems ranking second-highest) ranked higher for crop range utility.

Within the data-related factors, analysis ratings varied widely, and the types of analysis developed for the systems included calibration, trait extraction, 3D model reconstruction, plot extraction, among others. While each system included some level of analysis in their work, none of the selected FB-HTP projects included the post-processing code or tools as Supplementary Materials made available with publication; however, in some cases supplementary data were made available [15,25]. In a majority of the references, details about the methods, and in some cases the equations used for trait measurement, were described in detail [21,24,26], while some systems included very little detail about the trait extraction methods [15,16,23]. Accuracy and precision across most systems were, in general, highly suitable for phenotyping applications [14,20,21,24,25]; some systems showed lower correlations between multiple methods for measuring the same phenotypes, but that is to be expected as each method has some variability. Additionally, even studies which included ground truth data were expected to have some variability due to human error. Finally, for dealing with environmental variability (namely, solar radiation and changes in lighting conditions), a wide range of strategies were evident, including completely enclosing the sensors to control the data acquisition environment [14,24], adding sensors to measure changes in lighting or solar radiation [23,25], and not accounting for variability in environmental conditions [16,19,20,22]. Some platforms limited the time window for data collection to ensure more optimal conditions [15,21], which is also a strategy, but limits the usability of the system. Additionally, the need to actively consider environmental conditions at the time of data acquisition depends on the phenotypes of interest, because solar radiation and sunlight have less of an effect on physical plant architecture measurements, but a potentially significant effect on reflectance, temperature, and color measurements.

**Figure 6:**
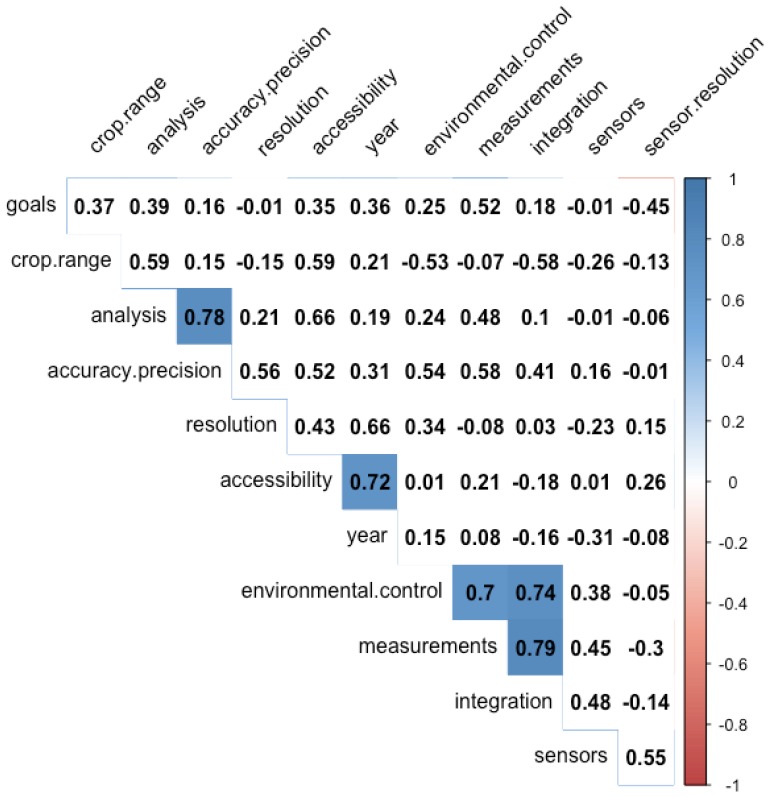
The intercorrelations between the utility scores for the subsystem factors. Only scores that were significant at the p<0.05 level were shaded in the matrix. Note that the system from [16] was not included in these data due to missing data points for multiple factors (see Table 7).

**Figure 7:**
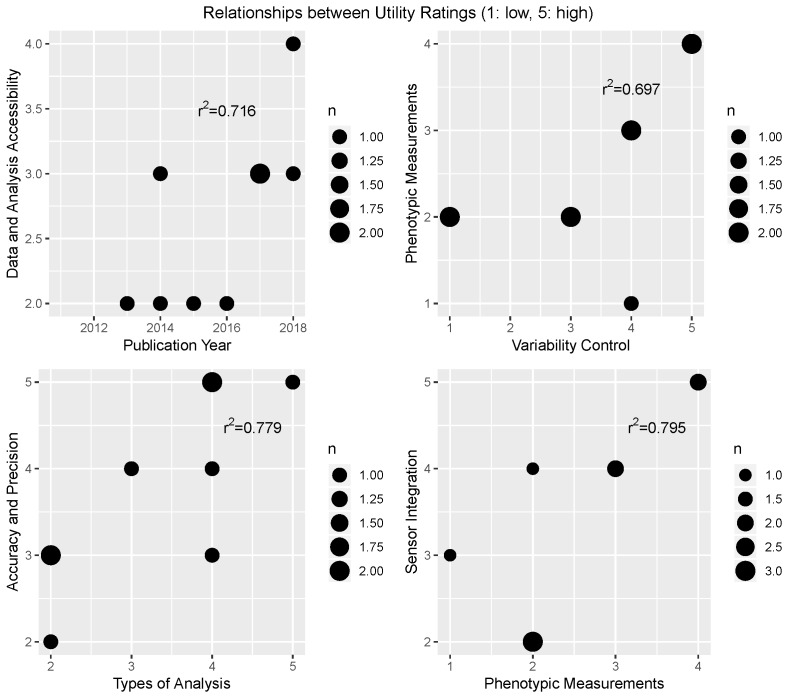
Scatter plots for the significant utility correlations from the analysis presented in Figure 6: Accessibility and publication year ($r^{2}=0.716$); environmental variability control and phenotype measurements ($r^{2}=0.697$); accuracy and precision and analysis types ($r^{2}=0.779$); and phenotype measurements and sensor integration ($r^{2}=0.795$).

A correlation matrix for the utility ratings is shown in Figure 6, with only the significant correlations (p<0.05) highlighted. The Spearman correlation coefficients are shown in the matrix as well. The data for the significant relationships are shown in scatter plots in Figure 7. These correlations highlight several interesting trends observed in these data. First, there is an increasing trend between publication year and the accessibility of the data and analytics methods, indicating that the trend of open source software and hardware in research is possibly increasing. Second, there is an increasing relationship between the number of phenotypic measurements and the environmental variability control; this is likely due to the fact that many of the platforms that controlled for lighting conditions did so by enclosing the sensors in fabric or including sensors for measuring ambient lighting; both strategies that were completed on sensor carts which can hold more sensors and make more measurements compared to small, portable lightweight robotic systems. Third, there was an increasing relationship between the types of analysis included in the study and the reported accuracy and precision of the system. This may be because more data-intensive studies implemented ground-truth methods and focused on the analysis as much as they did on the platform development. Finally, there was an increasing relationship between the ability to integrate new sensors, and the possible phenotypic measurements. The systems with high utility ratings for integration designed the systems to be modular from the beginning, with an emphasis on flexibility for future sensors; these systems also tended to use sensor carts capable of recording a larger number of possible recorded phenotypes.

#### 3.3.3. Total Complexity and Utility Scores

**Figure 8:**
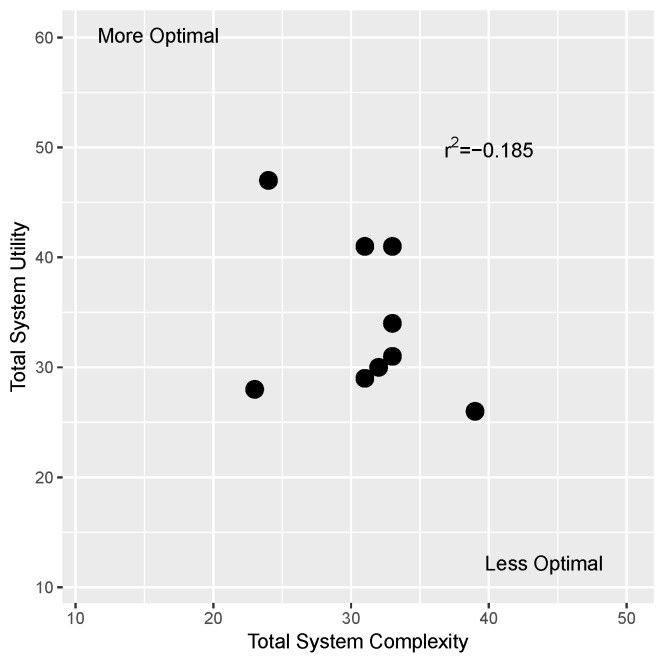
Scatter plot of the total utility scores and total complexity scores for each system. The most and least optimal regions on this graph are also highlighted.

The total complexity and utility scores were calculated and plotted for each system, as shown in Figure 8. While the correlation between utility and complexity total scores is weak (Spearman correlation coefficient $r^{2}=0.185$), there are some systems that have more desirable total scores compared to others. A more ideal system would have a high utility rating and a lower overall system complexity rating; the system developed in [24] is closest to this more optimal relationship. Alternatively, a system that ranks higher in overall complexity and lower in total utility is less desirable; the platform in [22] is closest to this less optimal location. One major difference between these two cases is the development of software in [24] that enables a more modular, flexible system with an easy-to-use GUI that facilitated automated data collection, as opposed to the system in [22] that was hardware-driven with very little software and no user interface; however, this is also an illustrative example of how these systems can improve over time, as the sensor system in [22] was one of the first multi-sensor phenotyping systems developed for high-throughput field work, paving the way for the development of future systems.

## 4. Discussion

This framework acknowledges that there are multiple approaches to designing field-based phenotyping systems, that every team and project have different goals, that there are multiple paths to achieve satisfactory results, and that the definitions of satisfactory results vary between projects and systems. While this framework is agnostic to some of these factors in order to compare across different case studies, there are trends in the complexity and utility rating data and how the subsystems interface that illustrate several observations and areas for improvement in the current FB-HTP development from which future research directions can be highlighted.

### 4.1. Observations from Complexity and Utility Rating Data

Several observations can be made from the complexity and utility score assessments of the FB-HTP systems, which include: (i) lack of information and development related to data handling, transfer, and storage; (ii) underdeveloped HMI for phenotyping system operators; and (iii) competing contributions from factors towards the total system complexity and utility scores.

#### 4.1.1. Observation 1: Lack of Included Information Related to the Data Subsystem

Data handling and processing remains a challenge for the phenomics community. Phenotypic data are nearly infinite in spatial and temporal scales, vary greatly depending on the project, and require significant storage capacity [3,27]. These challenges are evident when assessing the complexity scores for the data-related factors. A few case studies were methodological in developing raw data handling methods to automatically organize data into a database and process metadata in real time [14,15,16]; however, for many case studies, the data were collected individually for each sensor and not managed effectively until the post-processing phase. Additionally, many systems did not detail their methods for trait data management through the post-processing pipeline [21,23,24,25], and for the systems that did mention their data handling methods, some level of manual data handling was required [19,20,22].

The manual transfer of data from the field to a central server for post-processing is both a physical and temporal bottleneck in the efficiency of FB-HTP systems and a problem inherent to human-data interaction (HDI). HDI can be defined as the human manipulation, analysis, and sensemaking of data [28]. The data manipulation aspect is of particular significance to the development of FB-HTP, and in general has the following challenges [28]:

- **Large scale**. Datasets are on the order of thousands, millions, or even billions of items.
- **Unstructured**. The data are often heterogeneous and lack coherent structure.
- **Multiple sources**. The data must be combined and synthesized from several different sources, each with their own data format and possibly without explicit relations.

These challenges directly impact the data handling and manipulation aspects of HDI. Maintaining data management strategies and automating as much of the pipeline as possible will be necessary for future FB-HTP systems. Additionally, it is critical that current efforts report on their data handling and management methods so the research community can learn and make progress as a whole. Moving forward, one particularly important research area will be reducing or eliminating the amount of manual interaction required to transfer data from the field, which will likely require some amount of data reduction. Potential areas of research in this domain include edge computing and on-board data processing to extract and upload only trait data, rather than uploading entire data sets; however, these are viable solutions only after specific traits of interest are identified and raw data are no longer needed. As the research community continues to relate phenotype to genotype with increasing efficiency, data reduction is expected to play a central role in future efficient deployments of FB-HTP technology.

#### 4.1.2. Observation 2: Inadequate Human-Machine Interaction Technology

Another trend from the system complexity analysis is the development and implementation of effective interfacing, or HMI, for phenotyping operators and system monitors. Several of the case studies did not include the development of a dedicated user interface [16,20,25]. Some systems mentioned hardware or software interfaces [22,23]; however, these tend to require detailed knowledge of how the system operates to be used effectively. Only half of the case studies developed custom GUIs specifically for non-expert users [14,15,19,21,24], which included information such as sensor status indicators, vehicle status, and sample imagery. While this information is likely useful, there remains a lack of focused studies to determine the most effective HMI for field-based phenotyping systems.

Poorly designed HMI can cause errors in operation, which can affect the outcome of the phenotypic data collection. This is especially important when commercial field-based systems are eventually developed and selection decisions will be made from the collected phenotypic data. In general, manual operation of multiple phenotyping tasks is not desired as the perceptual and motor requirements of each task can interfere with each other and affect performance [29]; however, the decision on what tasks to automate is complex, and fully automating processes is not guaranteed to reduce cognitive load and fatigue [30]. The level of phenotyping system automation, which varied widely across these case studies, will ultimately affect the operator requirements and the determination of appropriate HMI [31]. A rich history of literature exists on designing automation around user requirements, and phenotyping or agriculture-specific models should be developed to assess which tasks should be automated [31,32]. First, however, there is a need for focused HMI studies to understand phenotyping operator requirements in a field setting, which includes the manual operation of machinery (e.g., tractors) and monitoring autonomous systems (e.g., small robots).

#### 4.1.3. Observation 3: Competing Drivers of System Complexity and Utility

As with any optimization problem, there are competing interests when designing systems that maximize utility while minimizing complexity. For example, in an effort to develop a system that can measure a large variety of phenotypes, a user may wish to increase the number of sensors; however, increasing the number of sensors adds technical complexity, as these sensors must be synchronized and controlled (autonomous or manually) based on specific user needs. Another example may be designing a platform that is highly versatile and can be deployed in a wide range of crop systems; however, this may require more resources, such as building a custom between-row robotic system or sensor cart, as opposed to mounting sensors to a piece of equipment readily available at the field site, such as a tractor. Evidently, there is a wide variety in factor complexity ratings, which each contribute to the total system complexity, but by standardizing the design of factors that can be controlled and homogenized across systems and environments (e.g., data storage, data handling, navigation), these factors can start to be eliminated as drivers of system complexity. It is also important to note again that complexity in this context is from the point of view of the user, and does not necessarily reflect the actual technical complexity of the system. Due to variability in factors that can be attributed to system complexity, a shift in focus may be required towards how the subsystems interact and function, as opposed to analyses at the individual factor level, which is discussed in the future directions section.

Ultimately, any additions and design modifications must be conducted strategically and effectively to ensure the system is usable by a wide range of users and levels of expertise. When it comes to the design and modification of FB-HTP systems, the framework presented in this work adds value and insight into the design process; in particular, utilizing the graphical relationship between total system utility and total complexity. When designing future FB-HTP systems, one can use the proposed rating system to approximate the complexity and utility scores and evaluate where the platform falls on the spectrum ranging from more optimal to less optimal, and how it compares to other existing platforms in the literature (as seen in Figure 8). This qualitative comparison can highlight factors that should be improved, including both factors that other systems have succeeded at optimizing (e.g., autonomous navigation) and factors that have yet to be consistently optimally designed within the larger phenomics community (e.g., data transfer and management).

## 5. Conclusions and Future Directions

To summarize, this work developed and implemented a framework for evaluating the complexity and utility of field-based, high-throughput phenotyping systems. The framework was developed by taking a systems-level approach to adapting previous work [5] and integrating the framework with established methods for complexity analysis [11,12]. A total of 10 case studies were selected and included in the framework implementation, which rated the complexity and utility of the individual factors and subsystems, as well as each system as a whole. The results from this evaluative framework can also be used to determine the overall optimality of a system design based on its total complexity and utility scores, which aids in identifying factors that need improvement in their design and effectiveness. This study suggests that more work is needed focused around the following areas: (i) data handling and management, specifically transfer from field to the rest of the pipeline, (ii) focused assessments related to human-machine interaction to facilitate usability across multiple users, and (iii) standardized design of factors common across all FB-HTP systems to limit the competing drivers of system complexity versus system utility. By improving the human-data interaction, such as implementing edge and on-board data reduction methods, the spatial and temporal bottleneck of transferring data from the field may be reduced. Additionally, focused studies and modeling of the human operator will enable better design of HMI technology that facilitates automated or semi-automated data collection while minimizing error. Finally, by selecting a subset of factors that can be standardized across nearly all FB-HTP systems, such as navigation and raw/trait data storage, these factors can be eliminated as major drivers of overall system complexity while maximizing system utility. This framework can be used to evaluate both previously developed and future proposed systems to approximate the overall system complexity level and identify areas for potential design improvement prior to implementation.

### Future Directions: Shifting Focus from Subsystems and Factors to Their Interfaces

**Figure 9:**
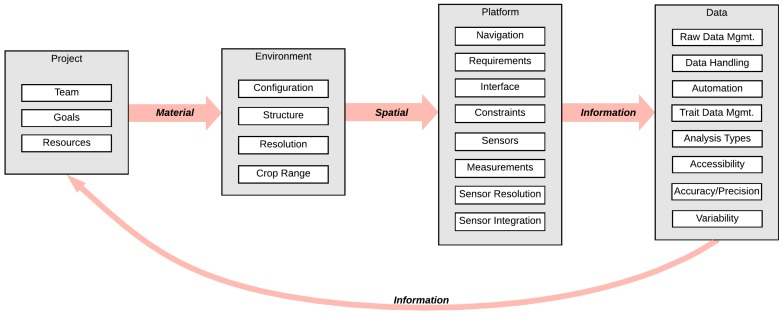
Subsystems, factors, and interface connection types of a FB-HTP system.

For a given phenotyping system, the project requirements dictate the operational environment and crop system, as can be seen from the goals described in Table 5. After these variables are defined, the platform is constructed based on the physical constraints of the crop and environment. Then, the development and capabilities of the platform are designed, which determine the type and amount of data that can be collected. Finally, the phenotypic data that are collected contribute to the overall project goal. This linear progression is illustrated in Figure 9. Ultimately, as the phenomics community progresses towards designing effective system factors that meet user requirements for complexity and utility, there remains the interfaces, or the connections, between sub-systems that can be optimized as well.

Interfaces between subsystems can be classified as four broad types: spatial, energy, information, and material [33], which are described in Table 8. The interface between the project and the environment is a *material* interface, which includes the physical inputs to create the operating environment of the phenotyping platform. In general, phenotypic experiments are highly controlled, planned operations that require material inputs to develop and maintain. The interface between the environment and the platform is *spatial* because the system needs to operate in spatial proximity to the plants and have a specific form factor to collect the required phenotypic data. The remaining two interfaces, between platform and data, and data and project, are *information* interfaces. The platform-data interface includes the transfer of raw data and other information from the field system to the data pipeline, and the data-project interface includes the transfer of phenotyping information and the biological and physiological meaning of the data to inform the project team members and contribute to the project goals.

By taking a systems approach moving forward, overall system performance can be improved by optimizing and standardizing the interfaces between the subsystems and treating the subsystems as black boxes. The material interface for experiment implementation has in general been standardized, but areas of future work include focusing on the spatial and information interfaces. Examples of this include designing a multipurpose physical system that can operate in many different crops, and standardizing the metadata and minimum data requirements for phenotyping experiments [27]. This approach is useful moving forward because the details of each factor will vary greatly between projects, but a systems interface approach allows for design standardization and comparison across multiple heterogeneous systems. By focusing on the interfaces between subsystems, interoperability and transferability across systems can be ensured, while maintaining flexibility for system development at the factor level. This flexibility is critical as the individual needs of a given project can be highly specific, requiring the development of new factor designs.

## Figures and Tables

**Table 1:** Factors associated with the project subsystem and descriptions of their associated categories.

Factor	Description	Category		
		*Basic*	*Intermediate*	*Advanced*
**Complexity**				
Team	The personnel, institutions, and expertise that comprise the team members	Few team members; single-institution; same or related disciplines	Multiple team members; one to two institutions; multidisciplinary teams	Many team members (>10), multiple institutions; diverse teams across many factors (geography, country, discipline)
Resources	The equipment (sensors and platforms), land, budget, and human resources available for use in the project	Small plots; minimal equipment; few team members actively participating in data collection	Mid-sized field trails; on-site equipment available; some team members dedicated for phenotyping	Large-scale plots; technically advanced sensors and equipment; many team members involved with data collection operations
**Utility**				
Goals	The high-level goal of the phenotyping project as stated in the introduction or motivation sections	One-dimensional, highly specific goal	Multi-dimensional, high-level goal(s)	Highly complex, multi-dimensional goal; multiple stated goals

**Table 2:** Factors associated with the platform subsystem and descriptions of the associated categories.

Factor	Description	Category		
		*Basic*	*Intermediate*	*Advanced*
**Complexity**				
Navigation	The navigation system capabilities of the phenotyping platform	Fully autonomous; little to no human interaction required; human as system monitor	Some autonomous functions; some level of human interaction required	Manual operation; no autonomy; continuous human input required for operation
Requirements	Operational requirements that must be met for the system to properly function	Highly flexible operational environments; few to none strict requirements	Moderate operational requirements; some strict, some flexible	Strict set of operating conditions and requirements
		***Basic***	***Intermediate***	***Advanced***
Interface	The user interface that controls the system during operation	Expert knowledge of the syste is required to operate using interface, for example, command line or hardware interfacing	Interface is operable with some training, requires some domain knowledge.	Visual Graphic User Interface (GUI) that enables operation by a non-expert
Constraints	The environmental and physical constraints for system operation	Can operate in a wide range of environments and settings	Can operate in a few environments and settings	Strict operating constraints and environments
**Utility**				
Sensors	The number and type of sensors on board used for phenotyping	Few or one sensor on board; independent sensor operation	Few sensors on board; integration of sensors begins to occur	Many sensors on board; full system integration and synchronization required
Measurements	The types of phenotypic measurements that the system enables during data collection	One to few measurement types; multiple measurements can come from one sensor	Multiple traits of interest; data from a few sensors may be combined of synthesized	Complex traits of different types (e.g., physical, spectral); measurements are integrated to produce additional metrics
Resolution	The resolution of the sensor(s) included in the system	Low-resolution data; point data only; or sensors only capable of collecting plot-level data	Sensors are capable of producing reasonably high resolution data at the plant level	Multiple high-resolution sensors
Integration	The ability to add additional sensors to the platform for custom configurations	System was constructed specifically for one or few types of individual sensors; highly application specific	System is not ready for integration with any sensor type, but new sensors can be added with some effort	System was constructed in a modular fashion with flexibility and sensor integration in mind

**Table 3:** Factors associated with the environment subsystem and descriptions of the associated categories.

Factor	Description	Category		
		*Basic*	*Intermediate*	*Advanced*
**Complexity**				
Configuration	The planting configuration of the crop system, including row spacing, plant height, and field layout	Wide row spacing; low crop density; symmetric and ordered layout	Narrower row spacing; taller crops; more complex layout	Tallest crop height; dense planting; narrow row spacing; complex field layout
Structure	The physical characteristics of plant architecture, including aspects that impact measurement	Simple plant morphological structure; few occlusions; high visibility	More complex morphological structure; some occlusions	Complex morphological structure; many occlusions
**Utility**				
Resolution Range	The range environmental resolution of the phenotypic data	Coarse, plot-level data	Row-level data; aggregate plant-level data	Plant or plant organ data; single plant per genotype
Crop Range	The range of crop systems that the platform is capable of operating in	Platform built for and operates in one specific crop	Platform is moderately flexible and can operate in a broad category of crop	System is highly flexible and can operate in a wide range of crops

**Table 4:** Factors associated with the data subsystem and descriptions of the associated categories.

Factor	Description	Category		
		*Basic*	*Intermediate*	*Advanced*
**Complexity**				
Raw Data	The methods for storing and organizing the raw data collected from the sensors	Data automatically organized according to date, time, and location	Some organization or metadata recorded automatically, but some manual processing needed	All organization and metadata handling manually required post-data collection
Data Transfer	How the data are handled throughout the processing pipeline	Processing on-board with automatic transfer for storage; little to no manual handling	Some manual data handling required; most of the process is automated	Complete manual data transfers required for each step of the pipeline
		***Basic***	***Intermediate***	***Advanced***
Processing Automation	The level of autonomy in the data post-processing pipeline, after data transfer from the platform	Fully autonomous post-processing software or techniques used to extract the trait data	Semi-automated or semi-supervised post-processing techniques used for trait extraction	Trait extraction methods were fully manual or required continuous human input
Trait Data	The methods for storing and organizing the trait data after computation	Data automatically organized, usually into a database, with accompanying metadata	Some automatic organization and metadata recording, but some manual processing needed	All organization and storage handled manually after computation
**Utility**				
Analysis	The type of analytical solutions that are made available with the system for post-processing of the data	Basic analysis of traits; raw trait data used without analysis	Some post-processing techniques made for trait extraction; Some detail provided about the analysis methods with reference to softwares used	Ability to extract a wide range of phenotypic traits; Details explanation provided
Accessibility	The accessibility and availability of the data processing methods	No code or scripts made available; no reference to software used, or proprietary software used only; no details about the methods provided	Open source software used; methods are standard or available, but specific code used was not provided	Open source software used, and code or scripts used for analysis made available for use
Accuracy & Precision	The accuracy and precision of the resulting processed phenotypic data	Relatively low accuracy and precision; no ground-truth procedures performed	Moderate agreement between system measurements and ground truth data	Ground-truth results presented for phenotypic trait analysis, resulting in high accuracy (> 90%)
Variability	How the system handles variability in environmental conditions in the resulting data	Environmental variability was not controlled for or measured	Variability in environmental conditions was measured with each sensor measurement	Attempts were made to control environmental variability for all sensor measurements

**Table 5:** Basic information for the selected case studies, including platform, crop system, institutions, and goals of developing the system.

Ref(s)	Author Institutions	Goals	Platform	Crop
[14]	Univ. of Applied Sciences Osnabrück (Competence Centre of Applied Agricultural Eng.); Universität Hohenheim (State Plant Breeding Institute, Institute of Agricultural Engineering); AMAZONEN-WERKE H.Dreyer GmbH & Co. KG	To develop a tractor-pulled multi-sensor phenotyping platform for small grain cereals	Tractor-pulled sensor trailer	Small grains and cereals (tested in triticale)
[15]	Julius Kühn-Institut, Federal Research Centre of Cultivated Plants; Liebniz Institute for Agricultural Engineering Potsdam-Bornim; University of Bonn, Department of Geodesy; Geisenheim Uni., Dept. of Viticultural Engineering	To develop an automated phenotyping platform to screen for phenotypic traits on a single-plant level in a reasonable time	Autonomous chain vehicle	Grapevines
[16,17,18]	Robert Bosch GmbH; University of Applied Sciences Osnabrück; AMAZONEN-WERKE H.Dreyer GmbH & Co. KG	To develop an autonomous field scout robot for phenotyping at the single plant level	Autonomous four-legged rover	A wide range of row crops, including maize
[19]	CSIRO Plant Industry and Climate Adaptation Flagship, Computational Informatics, and High Resolution Plant Phenomics Centre	To develop an autonomous platform and a software workflow solution for plot-based data	Autonomous unmanned aerial vehicle	Row crops that require plot-level data (e.g., sorghum, sugarcane)
[20]	University of Illinois at Urbana-Champaign (Civil and Environmental Engineering); Iowa State University (Agricultural and Biosystems Engineering); Massachusetts Institute of Technology	To image the plant from both the side and above and enable phenotyping throughout the entire growing season	Portable between-row robot	Energy sorghum
[21]	Iowa State University (Agronomy, Agricultural and Biosystems Engineering)	To create a self-propelled platform adaptable to tall crops	Modified tractor system	Tall biomass crops (e.g., sorghum)
[22]	University of Arizona (Agricultural and Biosystems Eng.); US Department of Agriculture, Arid-Land Agricultural Research Center; Cornell University (Plant Breeding and Genetics); Rothamsted Research (Plant Biology and Crop Science)	To develop a system that records multiple types of data in a single pass to increase throughput and enable more accurate and comprehensive specification of phenotypes	Proximal sensing cart	Cotton
[23]	University of Nebraska-Lincoln (Biological Systems Engineering; Agronomy and Horticulture)	To develop a multi-sensor system to collect high throughput, plot-level trait measurements for plant breeding	Proximal sensing cart	Soybean and wheat
[24]	University of Georgia (Electrical and Computer Engineering, Agricultural and Environmental Sciences, Arts and Sciences)	To develop and evaluate a FB-HTP system accommodating high-resolution imagers	Sensing system integrated into a high-clearance tractor	Cotton
[25]	University of Missouri (Electrical Engineering and Computer Science, Division of Plant Sciences)	To develop a ground vehicle that measures individual plants coupled with an observation tower that oversees an entire field	Autonomous mobile platform and stationary tower	Maize and sorghum

**Table 6:** Scoring results for the complexity factors after applying the framework. The ratings were reported on the following five-point scale from a user perspective: 1 = *Basic*, 2 = *Basic-Intermediate*, 3 = *Intermediate*, 4 = *Intermediate-Advanced*, and 5 = *Advanced*, where a lower rating for each complexity factor is desirable.

Refs.	[14]	[15]	[16,17,18]	[19]	[20,26]	[21]	[22]	[23]	[24]	[25]
**Project**										
Team Members	5	5	5	4	1	2	4	2	2	1
Resources	4	2	4	4	2	3	3	2	4	5
**System**										
System Navigation	4	2	2	1	2	2	4	5	4	3
Operation Requirements	3	2	1	4	3	4	2	1	1	2
User Interface	2	1	4	4	5	2	5	1	1	5
Operation Constraints	3	3	2	5	4	4	4	5	2	4
**Environment**										
Field Configuration	3	2	2	1	4	2	3	2	1	2
Crop Structure	3	2	3	2	4	4	1	1	2	3
**Data**										
Raw Data Management	1	1	1	3	3	3	4	4	3	3
Data Handling	2	1	2	2	3	3	4	3	3	3
Data Processing Automation	1	2	3	3	2	3	5	5	1	2
Trait Data Management	2	2	1	NA	1	NA	NA	4	NA	NA

**Table 7:** Scoring results for the utility factors after applying the framework. The ratings were reported on the following five-point scale from a user perspective: 1 = *Basic*, 2 = *Basic-Intermediate*, 3 = *Intermediate*, 4 = *Intermediate-Advanced*, and 5 = *Advanced*, where a higher rating for the utility factors is desirable.

Refs.	[14]	[15]	[16,17,18]	[19]	[20,26]	[21]	[22]	[23]	[24]	[25]
**Project**										
Project Goal	2	1	4	3	3	1	1	4	3	5
**System**										
Sensors	5	2	5	3	2	1	2	2	5	1
Phenotype Measurements	4	1	5	2	2	2	2	3	4	3
Sensor Resolution	3	4	NA	4	3	3	3	2	4	2
Sensor Integration	5	3	5	2	2	2	4	4	5	4
**Environment**										
Environmental Resolution	3	5	5	1	5	4	3	1	5	5
Crop Deployment Range	3	1	3	5	4	4	3	3	3	4
**Data**										
Types of Analyses	4	2	3	4	3	4	2	2	4	5
Analysis and Data Accessibility	2	2	1	3	3	3	2	2	4	3
Accuracy and Precision	5	3	NA	3	4	4	3	2	5	5
Environmental Variability	5	4	1	1	3	3	1	4	5	4

**Table 8:** Simple taxonomy of subsystem interactions [33].

Type	Description
Spatial	Associations of physical space and alignment; needs for adjacency or orientation between two elements
Energy	Needs for energy transfer/exchange between two elements
Information	Needs for data or signal exchange between two elements
Material	Needs for material exchange between two elements

## Supplementary Materials

The following are available online at https://www.mdpi.com/1424-8220/19/16/3582/s1.
