# Supplementary material for: A Framework for Evaluating Field-Based, High-Throughput Phenotyping Systems: A Meta-Analysis
Source: Sensors (Basel). 2019 Aug 17;19(16):3582. doi: 10.3390/s19163582 (PMC6720174; doi:10.3390/s19163582)
Supplement: Supplementary file 1 [file sensors-19-03582-s001.pdf]

1 Busemeyer et al., 2013

Table 1: Complexity framework application results.

Factor	Description	Score
**Complexity Scores**		
**Project**		
Team	6 engineers; 5 plant scientists; 1 industry personnel; representing 4 different institute affiliations	5
Resources	Field machinery (tractor); 25 x genotypes across 600 plots; technician for operation; advanced sensors	4
**Platform**		
Navigation	Manually pulled tractor cart; data acquisition is manually started at the beginning of each row and terminated at the end of each row	4
Requirements	Generator on tractor; operator to drive; availability of tractor or similar machinery	3
Interface	Industrial PC with a custom, dedicated GUI for data collection; manual steering for navigation	2
Constraints	Plant height up to 1.6 m with variable clearance; tractor width of 1.25 m (not described as variable width)	3
**Environment**		
Configuration	Uniform planting width; 1 row spacing of 1m; row length of 4m	3
Structure	Shorter, dense plants (cereal grains); many occlusions of individual stems and heads; light curtain images in the middle of the row (Fig. 2); other sensors image from a top-down view	3
**Data**		
Raw Data	Server on an industrial PC with MySQL database on platform for automated organization with accompanying metadata during collection; HSI sensor on a separate server located on the laptop; all data synchronized with an NTP server	1
Data Transfer	Manual transfer to stationary workstation for automated offline post-processing (inferred from Fig. 3)	2
Processing Automation	New raw data that are added are detected by the stationary workstation and automatically processed; trait extraction is automatic after a manual calibration process	1

Table 1 – continued from previous page

Factor	Description	Score
Trait data	Results from the post-processing steps are stored in a result-database using MySQL; results of trait determination and calibration are stored as Microsoft Excel files	2
	**Total Complexity Score**	**33**
	**Utility Scores**	
**Project**		
Goals	Develop a tractor-pulled multi-sensor phenotyping platform for small grain cereals, with a focus on the technological development of the system	2
**Platform**		
Sensors	2D time-of-flight, RGB camera, laser distance scanner, hyperspectral, and light curtain; integrated for data acquisition	5
Measurements	Height, density, leaf/plant segmentation, penetration depth from the top and side, spectral reflectance	4
Resolution	Low-res ToF sensor (50x64); millimeter resolution height measurements; high-resolution hyperspectral scanner (320 bands)	3
Integration	The sensor system has a modular structure, and adding an additional sensor requires an additional microcontroller for that specific sensor; system is flexible and enables data capture at different frame rates for each sensor	5
**Environment**		
Resolution	Row-wise data accessibility of the raw data; trait data during post-processing were aggregated to the plot level	3
Crop Range	System designed specifically for small grain cereals; multiple crops possible within that category	3
**Data**		
Analysis	Calibration; plot segmentation; trait specific models	4
Accessibility	No indication that the MATLAB post-processing packages are available as supplementary information; Methods for error and calibration were described, but not for all trait extractions	2
Accuracy and Precision	Mean relative error of repetition: 0.031; mean relative calibration error: 0.024; height correlation with reference values: 0.99.	5
Variability	An enclosure make of black canvas was used to avoid exposure to direct solar radiation during imaging and data acquisition	5
	**Total Utility Score**	**41**

2 Kircherer et al., 2015

Table 2: Complexity framework application results.

Factor	Description	Score
	**Complexity Scores**	
**Project**		
Team	3 engineering; 2 geo-science; 4 plant science; 2 additional personnel assisted with platform development; representing 5 different institutions	5
Resources	2,700 grapevines (970 accessions) available for field studies; basic sensors (RGB); custom-built platform	2
**Platform**		
Navigation	Autonomous navigation given a set of accurate GPS coordinates to follow collected from a survey prior to platform deployment	2
Requirements	Pre-survey of GPS coordinates of each vine; human operator available to act as the system monitor during operation	2
Interface	A custom GUI was developed that controlled the image transport and storage, enabled camera triggering, and allowed the operator to view the data and set camera parameters	1
Constraints	Time of imaging constrained to nighttime due to better lighting conditions; planting configuration wide enough for platform to fit in between the rows	3
**Environment**		
Configuration	Vineyard row spacing (not specified, but normally wider than row crops); vines have identifiable GPS coordinates; between-row navigation and imaging	2
Structure	Vines have a large form-factor; most of the fruit is readily seen from a side view (from Fig. 1 and 3), although there are some occlusions from the vine leaves	2
**Data**		
Raw Data	Collected on an industrial PC on board; utilizes a database (IMAGEdata, an institutional database system) as the data management system	1
Data Transfer	All images collected by the image acquisition software are imported directly into the IMAGEdata database with the associated metadata (plant ID, date and time)	1

Table 2 – continued from previous page

Factor	Description	Score
Processing Automation	Processing offline; color data automatically extracted into a .txt file, which is then imported into an SQL database; linear discriminant analysis performed using R afterwards	2
Trait data	Trait data are first stored in a .txt file and then transferred manually to an SQL database	2
	**Total Complexity Score**	**25**
	**Utility Scores**	
**Project**		
Goals	To automate the berry scoring process resulting in improved efficiency and reduced subjectivity	1
**Platform**		
Sensors	RGB/monochrome/NIR cameras; LED light bars	2
Measurements	Berry size and color	1
Resolution	Monochrome: 2448x2050; RGB: 2448x2050; NIR: 1388x1038	4
Integration	System is capable of adding in additional standard trigger cameras; does not seem readily available to add in additional novel sensor types (mentioned in future work, but not a current capability)	3
**Environment**		
Resolution	Per plant measurements of the following: berry count, size, and color (5 classes)	5
Crop Range	This platform was built specifically for phenotyping grapevines	1
**Data**		
Analysis	MATLAB tool for berry color extraction; R tool for color class prediction	2
Accessibility	The MATLAB tool developed for this specific platform was not made available for use; example dataset was published as supplementary material	2
Accuracy and Precision	Classification accuracy ranged from 70-97%, depending on the color class	3
Variability	Imaging was conducted at night with the light bars activated to control for different varying lighting conditions that occur during the day	4
	**Total Utility Score**	**28**

3 Ruckelshausen et al., 2009; Wunder et al., 2012; Bangert et al., 2013

Table 3: Complexity framework application results.

Factor	Description	Score
	**Complexity Scores**	
**Project**		
Team	5 industry personnel; 4 engineering; 2 agricultural science; representing 4 institutions	5
Resources	Panel of ongoing field trials with two cooperative partners; industry partners; advanced sensors	4
**Platform**		
Navigation	Fully autonomous system which uses RTK GPS and *a priori* information for localization; object detection provides robust backup for safety	2
Requirements	System monitor to input goals for path-planning	1
Interface	Navigation possible through a physical gamepad controller; Interface for sensor control not described	4
Constraints	Limited by chassis clearance for over-row imaging (0.8 m); leg design is flexible to accommodate a wide range of row spacings (0.75 to 2 m)	2
**Environment**		
Configuration	Planting configuration wide enough for platform to fit in between the rows (shown in maize); row width not specified, but platform width is variable	2
Structure	System was evaluated on maize plants early in the growing season	3
**Data**		
Raw Data	Stored on a MySQL database located on a central PC server on the system during data collection	1
Data Transfer	Manual transfer between the MySQL database to MATLAB processing pipeline assumed	2
Processing Automation	Raw data automatically organized into a database; level of automation for the MATLAB script was not specified	3
Trait data	Stored in a GIS database system OpenJUMP; each plant has its own database	1
	**Total Complexity Score**	**30**
	**Utility Scores**	
**Project**		

Table 3 – continued from previous page

Factor	Description	Score
Goals	Combine sensor systems with autonomous technology to develop a phenotyping system	4
**Platform**		
Sensors	3D ToF; NIR/VIS; hyperspectral; laser distance scanner; light curtain; RTK GPS	5
Measurements	Plant density; spacing; diameter; height; spectral reflectance; percent ground cover; biomass estimations; growth;	5
Resolution	Individual sensor models were not provided (only sensor types)	N/A
Integration	The system has a modular architecture where each sensor has its own microcontroller, enabling additional sensors to be easily integrated	5
**Environment**		
Resolution	Data collected at the individual plant level	5
Crop Range	Can accommodate a large range of row crops (limited by 0.8m chassis height clearance)	3
**Data**		
Analysis	Trait extraction in MATLAB; spatial visualization using GIS	3
Accessibility	Data processing techniques not described and MATLAB tools were not available; trait extraction methods not provided in detail	1
Accuracy and Precision	Ground truth data not collected and validation not performed	N/A
Variability	No sensors or techniques were used to eliminate variability in environmental conditions during data collection	1
	**Total Utility Score**	**32 + 2*NA**

4 Chapman et al., 2014

Table 4: Complexity framework application results.

Factor	Description	Score
	**Complexity Scores**	
**Project**		

Table 4 – continued from previous page

Factor	Description	Score
Team	10 members from CSIRO, including 6 in plant science/industry, and 4 in computational informatics; representing 1 institution	4
Resources	Six copters available for use; hundreds of plots across four crops were used in the studies	4
**Platform**		
Navigation	Autonomous flight paths are generated based on the user input of the region of interest (ROI)	1
Requirements	A certified pilot for operation; flight plan; sufficient weather conditions for operation	4
Interface	RC controller for manual flights or a user planning tool to generate autonomous flights; both require certification or expertise for operating unmanned aircraft	4
Constraint	Vehicle payload and flight time (battery); flight regulations	5
**Environment**		
Configuration	Wide range of plot designs; aerial survey of agricultural fields; top-down imaging	1
Structure	Can image a wide range of plat structures (tested on sorghum, wheat, and sugarcane); some occlusions occur at later growth stages	2
**Data**		
Raw Data	Stored on board in flash memory	3
Data Transfer	Manual transfer off the platform, then automatically organized into a directory based on location, date, and flight number for the day	2
Processing Automation	Automatic directory created when data is downloaded from platform; mosaics automatically processed; image data sets are individually analyzed afterwards in R (some traits are automatic, some are semi-supervised); likely manual transfer between steps and softwares	3
Trait data	Methods for storing and managing trait data were not discussed	N/A
	**Total Complexity Score**	**33+NA**
	**Utility Scores**	
**Project**		
Goals	Reduce the cost and required time of breeding trials	3
**Platform**		

Table 4 – continued from previous page

Factor	Description	Score
Sensors	Digital cameras; thermal camera; visible camera with red edge filter	3
Measurements	The following phenotypes were focused on: Ground cover; lodging; relative transpiration	2
Resolution	Thermal camera: 640x480; digital cameras: 3648 x 2736	4
Integration	System can only carry a few (2-3) sensors at one time; therefore, integration is possible by switching out existing sensors with different cameras to interface with the on-board computer	2
**Environment**		
Resolution	Plot level data; 10–20 mm resolution depending on flight altitude	1
Crop Range	Wide range of crops (aerial view - not restricted by any row spacings or plot configurations)	5
**Data**		
Analysis	DEM generation; plot extraction; spectral analysis	4
Accessibility	Commercial software used for DEM/mosaic creation; R software library was not made available with publication, although the software is open source	3
Accuracy and Precision	Ground cover: 0.78 correlation with estimated ground cover and 100 random ground-truth plant counts (no other measures of accuracy were included)	3
Variability	No sensors or techniques were used to eliminate variability in environmental conditions during data collection	1
	**Total Utility Score**	**31**

5 Young et al., 2018; Baharav et al., 2017

Table 5: Complexity framework application results.

Factor	Description	Score
	**Complexity Scores**	
**Project**		
Team	3 engineering (acknowledgements included 1 plant biology and field technicians); Research was conducted at one institution (authors represented 3 intuitions at publication)	1

Table 5 – continued from previous page

Factor	Description	Score
Resources	Hundreds of test plots of sorghum at two field locations; field technicians to assist with running trials; custom-built platform	2
**Platform**		
Navigation	Autonomous navigation using RTK GPS, but must be given a path of coordinates to follow	2
Requirements	Clear rows (no object detection and avoidance); GPS points of path or an operator for manual control	3
Interface	Manual navigation: RC; autonomous navigation: remote connection to on-board computer via laptop	5
Constraint	Battery power; row width; payload capacity	4
**Environment**		
Configuration	Between-row measurements of row crops; side imaging and bottom-up view imaging; some plots were lodged and obstructing	4
Structure	Tall, dense crops; many overlapping features	4
**Data**		
Raw Data	Stored on-board the system on an external hard drive	3
Data Transfer	Manual data transfer for each sensor type from field robot to a cloud-based data management system	3
Processing Automation	After data uploaded to Clowder, traits were automatically extracted from the image data	2
Trait data	Stored and managed using Clowder, a cloud-based data management service; uploaded to BETYdb, an open source database	1
	**Total Complexity Score**	**34**
	**Utility Scores**	
**Project**		
Goals	To accelerate the breeding process for the development of biofuels	3
**Platform**		
Sensors	Stereo camera; RGB hemispherical imaging; ToF infrared sensor	2
Measurements	Height; stem width; leaf area index	2
Resolution	RGB: 3840 x 2160; stereo: 752x480; ToF:176x120	3

Table 5 – continued from previous page

Factor	Description	Score
Integration	Each sensor was operated using their own SDK through the on-board computer and new software would be required to integrate additional sensors onto the platform	2
**Environment**		
Resolution	Plant level data; can aggregate data at the row or plot level	5
Crop Range	Any row crop with a minimum row spacing greater than 0.48 m, up to 4.88 m tall	4
**Data**		
Analysis	Trait extraction; Statistical analyses	3
Accessibility	Data processing methods were explained theoretically, but not made available	3
Accuracy and Precision	85-87% accuracy for plant height and stem width measurements when compared to ground truth data	4
Variability	No sensors or techniques were used to eliminate variability in environmental conditions during data collection; however, the sensors were robust to changes in environmental conditions	3
	**Total Utility Score**	**34**

6 Salaz Fernandez et al., 2017

Table 6: Complexity framework application results.

Factor	Description	Score
	**Complexity Scores**	
**Project**		
Team	2 engineering; 2 agronomy; multiple field personnel; represents 1 institution	2
Resources	Field technicians; sorghum accession panel (SAP) of 307 accessions; tractor; tractor operator available	3
**Platform**		
Navigation	Autonomous navigation using RTK GPS; the autosteer platform first completes a path, and then is able to complete that path autonomously	2
Requirements	Tractor system; operator on-board the tractor; lighting conditions between hours of 10a and 4p to avoid low solar elevation angles	4

Table 6 – continued from previous page

Factor	Description	Score
Interface	Computer software and a commercially available navigation interface	2
Constraint	Need set of GPS points *a priori* for navigation; max speed of 0.67 m/s due to camera buffer; fixed wheel-base width of 145 cm	4
**Environment**		
Configuration	Wide planting rows: 2.28 m; larger, taller plants (Fig. 7)	2
Structure	Tall crops up to 3m; some overlap between neighboring plants	4
**Data**		
Raw Data	Stored on a solid state drive (SSD) on-board the platform	3
Data Transfer	Data collected on a rugged laptop in the field; assume manual data transfer was required to relocate data to another server for processing; data consisted of one primary type (stereo image sets) with a descriptive naming convention	3
Processing Automation	Two data analysis approaches were discussed: one was semi-automated, and the other was fully automated; semi-automated algorithm has a GUI to assist the user in the post-processing steps	3
Trait data	No mention of how the trait data were ultimately managed and stored	N/A
	**Total Complexity Score**	**32+NA**
	**Utility Scores**	
**Project**		
Goals	To develop a HTP platform that can extract plant architecture traits and perform GWAS studies comparing results from automated vs. manually collected trait data	1
**Platform**		
Sensors	Up to three sets of stereo cameras	1
Measurements	Plant height and stem width; architecture reconstructions	2
Resolution	Each color camera has a resolution of 1624x1224	3

Table 6 – continued from previous page

Factor	Description	Score
Integration	Platform uses an on-board laptop to run the acquisition software, readily able to integrate with sensors that can be integrated with the FlyCapture SDK (e.g., Point Grey sensors); otherwise, new software would be required	2
**Environment**		
Resolution	Automated trait extraction method collects height data at the row level; supervised trait extraction method can obtain measurements at the individual plant level	4
Crop Range	Row crops with the appropriate row spacing such that the tractor system fits; can image a wide range of crop heights up to 3 m	3
**Data**		
Analysis	Dense reconstruction; trait extraction	4
Accessibility	Algorithms were not made available in supplementary materials, but details regarding each trait extraction approach were included in the manuscript	3
Accuracy and Precision	0.75-0.93 correlation between automated/semi-automated phenotype measurements and ground truth manually collected data	4
Variability	Imaging was performed between the hours of 10 a-4 p to avoid undesirable sun angles towards the sensors; no sensors were used to measure ambient environmental or lighting conditions	3
	**Total Utility Score**	**30**

7 Andrade-Sanchez et al., 2014

Table 7: Complexity framework application results.

Factor	Description	Score
	**Complexity Scores**	
**Project**		
Team	2 engineering; 6 USDA ARS personnel in the areas of plant science, genetics, and biology; representing 4 institutions	4
Resources	Irrigated field site in AZ; 25 cotton cultivars for imaging; tractor	3

Table 7 – continued from previous page

Factor	Description	Score
**Platform**		
Navigation	Manually driven tractor system	4
Requirements	Operators; pre-deploy to collect reference measurements for calibration	2
Interface	Hardware interface with the sensors using Campbell Scientific data loggers	5
Constraint	Constrained by chassis clearance and distance between the sonar instrument and plant canopy (max distance 1.4 m was not always tall enough to clear the tallest cotton plants completely)	4
**Environment**		
Configuration	Standard cotton row spacing; cultivars arranged into 200 plots with 1.02 m inter-row spacing	3
Structure	Plant structure was not considered as the sensors provided point measurements facing downwards	1
**Data**		
Raw Data	Stored on individual data loggers as electronic data files in their raw formats	4
Data Transfer	Manual transfer off of the data loggers would have been required prior to analysis	4
Processing Automation	Manual processing of the data; no automated software or pipelines mentioned	5
Trait data	No mention of how the trait data were ultimately managed and stored	N/A
	**Total Complexity Score**	**39+NA**
	**Utility Scores**	
**Project**		
Goals	To phenotype cotton plants (physiology and morphology traits) in both watered and stressed plants	1
**Platform**		
Sensors	Sonar; infrared radiometer; multispectral NDVI sensor	2
Measurements	Canopy height; canopy temperature; NDVI	2
Resolution	Sonar: millimeter-resolution height data; Multispectral: 10 mm resolution bandwidth; NIR filter: 60 nm bandwidth	3

Factor	Description	Score
Integration	Any new sensor would require another data logger and associated hardware and power source; no central computing system to tie into for individual control/triggering of new sensors	4
**Environment**		
Resolution	Sensors provide point measurements; Aggregated to the row or plot resolution data	3
Crop Range	Any row crop with the required row spacing up to a maximum clearance of 1.4 m	3
**Data**		
Analysis	Trait extraction; statistical analysis	2
Accessibility	SAS, a commercial proprietary software, was used for basic statistical analysis	2
Accuracy and Precision	The phenotyping platform achieved the following correlation with manual measurements: Temperature: 0.75-0.82; NDVI: 0.61-0.62; Height: 0.76-0.78	3
Variability	No sensors or techniques were used to eliminate variability in environmental conditions during data collection	1
	**Total Utility Score**	**26**

8 Bai et al., 2016

Table 8: Complexity framework application results.

Factor	Description	Score
	**Complexity Scores**	
**Project**		
Team	2 engineers; 3 agronomists; 1 institution	2
Resources	On-farm access to soybean and wheat breeding trials (240 plots of wheat, 120 plots of soybeans); personnel available to run the data collection	2
**Platform**		
Navigation	Manual push-cart	5
Requirements	2 operators available to push the cart; flat field	1
Interface	Computer LabVIEW program GUI with a 1-button "measure" function to trigger all sensors	1

Table 8 – continued from previous page

Factor	Description	Score
Constraint	Non-motorized; max speed approximately 0.5 acre/hour	5
**Environment**		
Configuration	Top-down measurements of shorter row crops; 3 m wide soybean rows; 1.5 m wide wheat plots (each with 4 rows)	2
Structure	Device measures ratios; plant structure does not have a big impact on the system performance	1
**Data**		
Raw Data	Stored on computer as individual files and .csv files	4
Data Transfer	Assume all analysis was conducted on the computer; possible manual transfer for storage after data collection	3
Processing Automation	Manual analysis after data collection (no details of any automation provided)	5
Trait data	Stored on computer; no mention of database or management system	4
	**Total Complexity Score**	**35**
	**Utility Scores**	
**Project**		
Goals	To improve plant breeding by collecting high-throughput, plot-level trait measurements	4
**Platform**		
Sensors	Ultrasonic; NDVI/solar radiation; thermal infrared radiometer; fiber optic; RGB cameras; GPS	2
Measurements	Canopy height; NDVI; reflectance; temperature	3
Resolution	Camera: 1920x1080; Temperature: ± 0.2 °C	2
Integration	Adding additional sensors requires new hardware; use of LabVIEW enables new sensors to be controlled centrally through the GUI	4
**Environment**		
Resolution	Plot-level data	1
Crop Range	Any row crop with appropriate row spacing and a clearance of 1m between the canopy and the platform	3
**Data**		
Analysis	Most data use directly (point measurements); Spectral analysis (ratios); Green pixel segmentation	2

Table 8 – continued from previous page

Factor	Description	Score
Accessibility	Little details about analysis procedures, software, or techniques were included	2
Accuracy and Precision	No ground truth data were reported; inter-correlations among the sensor data were high for some (¿0.90), but less for others (¡0.7); wide range	2
Variability	Accounted for solar radiation by including an upwards facing flux sensor	4
	**Total Utility Score**	**29**

9 Jiang et al., 2018

Table 9: Complexity framework application results.

Factor	Description	Score
	**Complexity Scores**	
**Project**		
Team	3 engineering; 1 agricultural science; 1 genetics; representing 1 institution	2
Resources	Small-scale field for operation; an available operator; tractor system; advanced sensors	4
**Platform**		
Navigation	Manually driven tractor system	4
Requirements	Operator who is trained appropriately	1
Interface	LabVIEW program/GUI on a laptop computer and data collection is manually triggered	1
Constraint	Tractor clearance between 1.06 - 1.83 m; Row width between 1.52- 2.29 m	2
**Environment**		
Configuration	Single-plant layout representing 23 genotypes	1
Structure	Top-down view for data collection, so single plant must be visible in the imaging structure; plant structure reconstructed given only a top-down view	2
**Data**		
Raw Data	Stored on laptop in the field on a SSD hard drive	3
Data Transfer	Manual transfer from field system to local server	3

Table 9 – continued from previous page

Factor	Description	Score
Processing Automation	Image processing was automated	1
Trait data	No mention of how trait data were ultimately stored or managed	N/A
	**Total Complexity Score**	**24+NA**
	**Utility Scores**	
**Project**		
Goals	Develop a modular and customizable system for phenotyping	3
**Platform**		
Sensors	RGB-D sensor; hyperspectral; thermal; GPS; weather station	5
Measurements	Projected leaf area; plant width; plant volume; temperature	4
Resolution	RGB-D: 512x414 (depth), 1920x1080 (color); Thermal camera: 640x480; Hyperspectral camera: 640 (spatial) x 236 (spectral) and between 2.2 and 6.8 mm/pixel data	4
Integration	A modular system is setup so sensors can be easily integrated into the LabVIEW program	5
**Environment**		
Resolution	Per-plant data collected for all phenotypes/traits	5
Crop Range	Any row crop that has adequate intra-row spacing and meets specifications of the tractor system; sensor bar height variable from 1.2-2.4m	3
**Data**		
Analysis	Calibration; trait extraction	4
Accessibility	Methods and equations used for analysis were described in detail; no code or software tools were made available with publication	4
Accuracy and Precision	Correlation between manual and system measurements were as follows: Depth, 0.992; Temperature: 0.999; Spectral sensor: RMSE ¡ 1 nm	5
Variability	A black cover and a separate light source were used to control for ambient lighting and reduce variability	5
	**Total Utility Score**	**47**

10 Shafiekhani et al., 2017

Table 10: Complexity framework application results.

Factor	Description	Score
	**Complexity Scores**	
**Project**		
Team	2 engineering; 2 plant sciences; representing 1 institution	1
Resources	Field trials of sorghum and maize with different row spacing configurations; specialty robotics platform (ground system and manipulator arm); advanced sensor	5
**Platform**		
Navigation	Semi-autonomous; must first be aligned with the row, then proceeds autonomously	3
Requirements	Operator available for platform guidance; min row spacing of 26 inches	2
Interface	Interface details were not explicitly provided; however, the system uses ROS, which enables either a remote laptop for control or a hardware remote controller	5
Constraint	Width of 24.6 inches; payload limitations of 165 lbs; power capacity	4
**Environment**		
Configuration	Maize and sorghum fields planted at 114 and 152 cm row spacings	2
Structure	Row crops earlier in the growing season	3
**Data**		
Raw Data	Data are stored on the on-board system	3
Data Transfer	No information about data transfer was included; assuming manual data transfer off of the platform is required	3
Processing Automation	3D reconstructions are automated using existing techniques; trait extraction from the reconstructions were manual and semi-automated	2
Trait data	No details about how the trait data were ultimately manager and stored were included	N/A
	**Total Complexity Score**	**33+NA**
	**Utility Scores**	
**Project**		

Table 10 – continued from previous page

Factor	Description	Score
Goals	Show that the architecture, sensors. and algorithms for imaging are an reliable, accurate, and a fast approach to HTP	5
**Platform**		
Sensors	Trinocular camera; environmental (temperature, humidity, light intensity)	1
Measurements	3D reconstructions; leaf area index; plant height; Photosynthetically active radiation	3
Resolution	Trioncular: 1280 x 960 pixels	2
Integration	Acquisition software built using ROS which has a modular structure that lends itself to integrating additional sensors, which would required their own ROS node and software development	4
**Environment**		
Resolution	Data collected at the plant level, aggregated to the row or plot levels	5
Crop Range	Any crop with row spacings large enough for the vehicle to pass through (0.67 m wide) with height less than the robotic arm	4
**Data**		
Analysis	3D reconstructions; calibration; trait extraction	5
Accessibility	Platform built on open source software; Data analysis techniques were referenced but little explanation was provided	3
Accuracy and Precision	Height: ¡0.5 cm error; LAI: 0.996 correlation with manual measurements	5
Variability	Ambient light sensors to account for variability in environmental lighting conditions	4
	**Total Utility Score**	**41**
